# Comparative study of the persistence of anti-HIV activity of deoxynucleoside HIV reverse transcriptase inhibitors after removal from culture

**DOI:** 10.1186/1742-6405-6-5

**Published:** 2009-04-22

**Authors:** Elijah Paintsil, Susan P Grill, Ginger E Dutschman, Yung-Chi Cheng

**Affiliations:** 1 Department of Pediatrics, Yale University School of Medicine, New Haven, CT 06520, USA; 2 Department of Pharmacology, Yale University School of Medicine, New Haven, CT 06520, USA

## Abstract

**Background:**

Most in vitro assays of drug potency may not adequately predict the performance in vivo. Methods to assess the persistence of antiviral activity of deoxynucleoside analogs, which require intracellular activation to the active metabolites that can persist in cells, will be important for designing dosages, combination regimens, and assessing treatment compliance. Using an HIV-IIIB/TZM-bl indicator cell culture system, we assessed the ability of an inhibitor to protect cells from infection and to delay viral rebound after removal of inhibitor from culture.

**Results:**

The order of protection of cells from HIV-infection was 4'-Ed4T > LFD4C > DDI > D4T > 3TC > AZT > FTC > NVP. The fold-increase in EC_50 _to delay viral rebound was DDI < 4'-Ed4T < LFD4C < FTC < D4T < 3TC < NVP < AZT. The ranking of persistence of anti-HIV activity of the inhibitors based on the two-component assay was DDI > 4'-Ed4T > LFD4C > FTC = D4T > 3TC > NVP > AZT.

**Conclusion:**

The persistence ranking was derived from assays based on measures of single viral replication-cycle and cumulative inhibition at multiple time-points. Therefore, a better indicator of the pharmacodynamic property of an inhibitor. The persistence of anti-HIV activity assay may complement in vitro potency assays to better predict in vivo performance of nucleoside analogs.

## Background

Nucleoside analog reverse transcriptase inhibitors (NRTIs) are the backbone of most highly active antiretroviral therapy (HAART) regimens. Most of the current HAART regimens consist of two NRTIs plus a non-nucleoside reverse transcriptase inhibitor (NNRTI) or a protease inhibitor (PI) [1]. NRTIs are phosphorylated to their triphosphate metabolites in the cells and compete with natural dNTP substrates for incorporation into HIV DNA leading to premature termination of the viral DNA chain elongation [2]. The active metabolites could persist in cells and the time of retention of the incorporated deoxynucleotide may vary with different analogs. The activity and toxicity of nucleoside analogs depend on both the concentration of the intracellular metabolites and the systemic pharmacokinetics. The clinical application of intracellular concentration is limited due to the technical difficulties with the quantification of intracellular concentration as well as the heterogeneity of cell populations. Hence, plasma concentration of inhibitors, which does not reflect the amount of active metabolites in target cells, has been used as surrogate for designing dosage and monitoring HIV therapy [3,4].

Peripheral blood mononuclear cells (PBMCs) are the natural target of HIV and therefore the ultimate host cells for HIV drug metabolism studies. However, in vitro use of PBMCs has several challenges; 1) lack of consistent susceptibility to HIV, 2) the need for stimulation of the cells that may affect the expression of cellular kinases and the dNTP pool size, 3) longer culture periods unfavorable for single-cycle assays, and 4) individual differences in PBMCs. Reporter systems have been used to overcome some of these challenges; they allow for the evaluation of HIV infectivity by using enzymatic reactions and demonstrate greater reproducibility with wider dynamic ranges [5-8].

The efficacy of a drug is predicted by its potency based on the inhibition of virus replication in cell culture over several days. The reliability of current measures of drug potency to predict in vivo performance has been questioned by several investigators [9,10]. Furgeson et al. argued that a single replication-cycle assay and measuring of cumulative inhibition at multiple time-points may be more robust pharmacodynamic measures [9]. Shen et al. proposed that the instantaneous inhibitory potential (IIP) based on the slope of the dose-response curve may better reflect clinical potency of a drug rather than the traditional measures like EC_50 _and inhibition quotient (IQ) [10]. In their assay, NRTIs had a slope of about 1, and only agents with slopes > 1 achieved high-level of inhibition of single-round infectivity [10]. Since the IIP is dependent on the slope of the dose-response curve it may not be sensitive enough to discriminate the differences in potency among the NRTIs that require intracellular activation for antiviral activity.

We recently reported a persistence of anti-HIV activity assay using HIV-IIIB/TZM-bl indicator cell culture system [11]. The TZM-bl indicator cell line is a HeLa cell line derivative that expresses high levels of CD4 and CCR5 along with endogenously expressed CXCR4 making it susceptible to both R5- and X4-tropic HIV viruses [12]. TZM-bl cells contain HIV LTR-driven β-galactosidase and luciferase reporter cassettes that are activated by HIV Tat expression. We compared the persistence of anti-HIV activity of a derivative of stavudine (D4T), 2',3'-didehydro-3'-deoxy-4'-ethynylthymidine (4'-Ed4T, Festinavir), to other analogs (AZT, D4T, and nevirapine [NVP]) [11]. AZT was more potent than 4'-Ed4T [13], however, the anti-HIV activity of 4'-Ed4T persisted longer than that of AZT after drug removal [11]. It was apparent that there was no correlation between the potency and the persistence of antiviral activity of an inhibitor. We have expanded our study to include other RTIs and to further investigate the apparent discrepancy between the potency and the persistence of antiviral activity of an inhibitor. In this study, we developed a two-component assay (i.e., protection of cells from HIV infection after drug removal and delay in viral rebound after drug removal). The two components are complementary and reflect the intracellular concentration and persistence of antiviral activity of an analog. We present the persistence of anti-HIV activity, a new pharmacodynamic parameter, which may complement other in vitro drug potency assays to better predict in vivo performance of nucleoside analogs.

## Methods

### Chemicals

4'-Ed4T was synthesized in the laboratory of Hiromichi Tanaka, School of Pharmaceutical Sciences, Showa University, Tokyo, Japan [14]. Elvucitabine (LFD4C) was synthesized in the laboratory of T. S Lin, Yale University School of Medicine, New Haven. Stavudine (D4T), zidovudine (AZT), didanosine (DDI) and nevirapine (NVP) were purchased from Sigma-Aldrich Corp. (St. Louis, MO). Lamivudine (3TC) and emitricitabine (FTC) were gifts from Triangle Pharmaceutical (Durham, NC). The purity of these compounds was verified by HPLC analysis. All other chemicals used were of analytical grade or higher.

### Cell lines and virus

The TZM-bl indicator cell line [12], obtained from J. Kappes through the AIDS Research and Reference Reagent Program, is a HeLa cell line derivative that expresses high levels of CD4 and CCR5 along with endogenously expressed CXCR4. Cells were cultured at 37°C in the presence of a humidified 5% CO_2 _atmosphere. The HIV-1 IIIB strain was received from Dr. John Mellors (University of Pittsburg).

### Assay for protection of cells from HIV infection after removal of drug from culture

**Figure 1:**
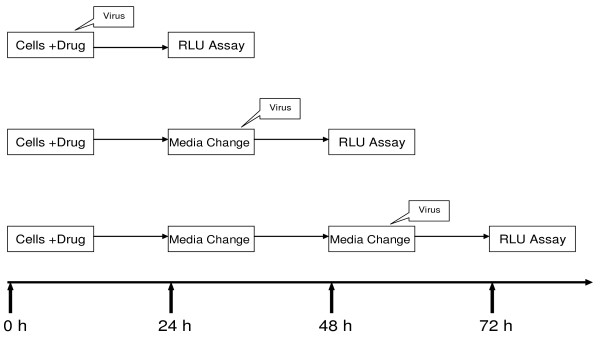
**Schematic representation of persistence assays**. (A) Assay for Protection of Cells from HIV Infection after removal of inhibitor, and (B) Assay for Viral Rebound after removal of inhibitor. RLU, is relative luciferase unit; wash implies the change of media without replacement of drug.

**Figure 2:**
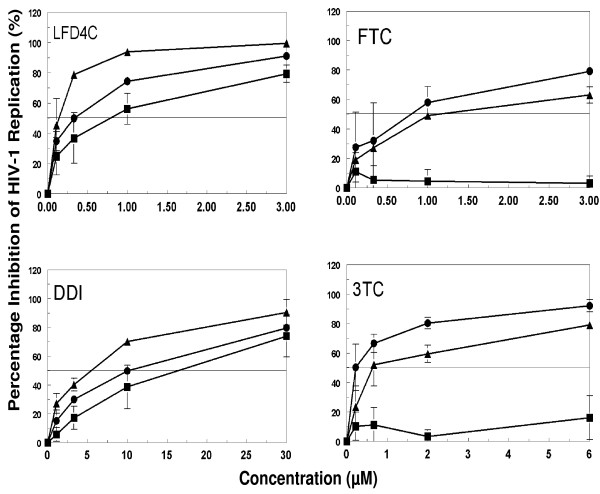
**Protection of cells from HIV infection after removal of inhibitor from culture**. The protection of cells from HIV infection was determined using a TZM-bl indicator cell line based-single cycle replication assay described in the "Materials and Methods" section and illustrated in Figure 1A. TZM-bl cells were cultured in the presence of various concentrations inhibitor (LFD4C, FTC, DDI, or 3TC) 24 h. The cells were then washed to remove extracellular drug, and infected with HIV-1 IIIB virus at an MOI of 0.1 at 0, 24, and 48 h of drug removal. The percentage inhibition of HIV-1 replication was determined by measuring the luciferase activity. The curves represent: percent inhibition of HIV-1 replication when cells were infected at the time of drug treatment and incubated together for 24 h (black circle); percent inhibition when cells were pre-incubated with drugs for 24 h and the media changed without replacement of drug prior to infection and incubated for 24 h (black triangle); and percent inhibition when cells were pre-incubated with drugs for 24 h and then the media was changed to remove drug at 0 and 24 h without drug replacement prior to infection (black square). Results are the average of at least three independent experiments. Error bars indicate standard deviations.

The schema for the assay for protection of cells from HIV infection is illustrated in Figure 1A; the details of the experiments have been previously published [11]. In brief, TZM-bl cells were plated at 5 × 10^3 ^cells per well in a 96-well microtiter plate in 100 μl of Phenol Red Free RPMI 1640 media and allowed to adhere for 15–18 h at 37°C prior to infection or drug treatment. After adherence of the cells, the media was changed and the cells were treated with various concentrations of 3TC, FTC, LFD4C, or DDI. Each drug concentration was replicated five times and the experiment repeated on at least three different occasions. To determine the effective concentration of inhibitor that inhibits 50% of viral growth (EC_50_), the cells were infected with HIV-1 IIIB virus at an MOI of 0.1 at the time of drug treatment (see Figure 1A, top panel). After 24 h of infection, the relative luciferase activity was determined as described below. The EC_50 _was calculated as the concentration of inhibitor that produced 50% of the relative luciferase activity of the control wells with HIV-infected cells in the absence of an inhibitor. For the protection of cells from HIV infection, a batch of plates was infected with HIV-1 IIIB virus after 24 h of incubation with drug and the drug removed without replacement (see Figure 1A, middle panel). A second batch of plates was incubated after replacement of media without drug for 24 h then infected with virus after a second change of media to remove any extracellular drug (see Figure 1A, bottom panel). Thus, the second batch of plates had two-24 h media changes without drug replacement prior to HIV infection. The cells in each well were harvested after 24 h of infection and lysed using luciferase assay reagent (Promega, Madison, WI). Firefly luciferase activities were quantified using a dual-luciferase reporter assay system (Promega, Madison, WI), and a microplate luminometer (FARCyte™, Amersham Biosciences Co., Piscataway, NJ). Background luminescence was determined from uninfected cells and subtracted from all experimental wells. Protection of cells from HIV infection was measured as a function of inhibition of viral replication (% of control replication without drug), calculated by dividing the mean number of luciferase units at each concentration of a drug by the mean number from cells containing no drug. The ability of a drug to protect cells from HIV infection is illustrated on plot with percentage inhibition on the *y*-axis against drug concentration on the *x*-axis (Figure 2).

### Assay for viral rebound after removal of drug from culture

The schema for the assay for viral rebound after removal of drug from culture is illustrated in Figure 1B. To evaluate viral rebound after drug removal, TZM-bl cells plated at 5 × 10^3 ^cells per well, as described above, were pre-incubated with various concentrations of inhibitor for 12 h at 37°C. Each drug (i.e., 4'-Ed4T, D4T, AZT, DDI, 3TC, FTC, LFD4C, or NVP) was tested at multiplicities of the EC_50_. Each drug concentration was replicated five times and the experiment repeated on at least three different occasions. The pre-treated cells were then infected with HIV-1 IIIB virus at an MOI of 0.05 at time zero and incubated at 37°C (Figure 1B). For the positive control experiment, i.e., EC_50 _in the presence of drug, the cells were harvested for the determination of luciferase activity after 24 h of incubation (see Figure 1B, top panel). For the plates designated for the rebound assay, the culture media were changed to remove extracellular drug without replacement of drug at 24 and 48 h post-infection (see Figure 1B, middle and bottom panels). At each time point, cells from a batch of the plates were harvested and the luciferase activity was determined as described above. Viral rebound was determined as a function of luciferase activity similar to that of day one of the untreated virus control.

## Results

### Efficacy of HIV reverse transcriptase inhibitors

Traditionally, in vitro efficacies are based on cumulative inhibition of multiple HIV replication cycles. We determined the efficacies of HIV reverse transcriptase inhibitors in a single HIV-replication cycle assay using TZM-bl indicator cell line. TZM-bl cells were infected with HIV-1 IIIB virus at an MOI of 0.1 in the presence of test inhibitor (L-FD4C, 3TC, FTC, or DDI) to assess the potency of each inhibitor. The EC_50 _in TZM-bl cells was determined after 24 h incubation by measuring the luciferase activity as previously described [15]. We next investigated the EC_50 _with pre-incubation of drug. The EC_50_s obtained with pre-incubation of cells with inhibitor (4'-Ed4T, D4T, AZT, DDI, 3TC, FTC, LFD4C, or NVP) 24 h prior to infection are shown in Table 1. EC_50_s 4'-Ed4T, D4T, AZT, and NVP have been previously published [11]; they were included in Table 1 for comparison. The EC_50_s obtained with pre-incubation of cells in our assay were more consistent with published potencies of these analogs based on multiple HIV replication cycles [16-19]. To our surprise, the EC_50_s of NVP and AZT increased by 3- and 4-folds, respectively, with pre-incubation. However, the potencies of 4'-Ed4T, DDI, and LFD4C improved with pre-incubation of the cells with the Inhibitors prior to infection. Therefore, pre-incubation of cells with nucleoside analogs may be necessary for assessing antiviral potency in single cycle HIV-replication assay.

**Table 1:** Antiviral activity of HIV inhibitors with or without pre-incubation of TZM-bl cells in single-cycle assay

**Inhibitor**	^**a**^**EC**_**50 **_**without pre-incubation of cells (μM)**	^**b**^**EC**_**50 **_**with pre-incubation of cells (μM)**	**Fold-Change in EC**_**50 **_**with pre-incubation** (^a^EC_50_/^b^EC_50_)
4'-Ed4T^c^	1.03 ± 0.45	0.37 ± 0.15	2.8
LFD4C	0.34 ± 0.02	0.16 ± 0.08	2.1
DDI	10.2 ± 1.1	5.4 ± 0.8	1.9
D4T^c^	1.08 ± 0.12	1.30 ± 0.21	0.8
FTC	0.80 ± 0.04	1.03 ± 0.23	0.8
3TC	0.40 ± 0.02	0.53 ± 0.01	0.8
NVP^c^	0.020 ± 0.004	0.063 ± 0.04	0.3
AZT^c^	0.018 ± 0.007	0.074 ± 0.040	0.2

^a ^Concentration required to inhibit HIV-1 replication in TZM-bl cells by 50% without pre-incubation of cells with inhibitor.

^b ^Concentration required to inhibit HIV-1 replication in TZM-bl cells by 50% when cells were pre-incubated with inhibitor 24 h prior to infection.

^c ^The EC_50 _of these analogs has been previously published [11]

Results are the average of at least three independent experiments.

### Protection of cells from HIV infection after removal of drug from culture

We investigated the persistence of anti-HIV activity to protect cells from HIV infection after removal of drug from culture. TZM-bl cells were pre-incubated with inhibitors to ensure that all inhibitors have reached their peak intracellular concentrations prior to HIV infection; this is based on our previous report that AZT reached its peak concentration at 2 h while d4T and 4'-Ed4T reached peak concentrations at 12 h and other published studies [11,20]. After 24 h of infection, the percentage of HIV-1 replication inhibition was determined by measuring the luciferase activity [15]. When infection occurred after the first change of media to remove extracellular drug (i.e., Figure 1A, at 0 h after removal of inhibitor), the inhibition curves for all the analogs shifted to the right except for L-FD4C, and DDI (Figure 2). When infection occurred after two consecutive changes of media without replacing drug (Figure 1A), only LFD4C, and DDI crossed the EC_50 _line; the other inhibitors tested did not protect the cells from HIV infection (Figure 2). None of the inhibitors currently in use for HIV-infected individuals tested protected cells from infection beyond 24 h of removal of drug at the highest concentration, except for DDI [11]. All the inhibitors showed a dose-dependent inhibition of viral replication (Figure 2). On the average, the ability of the inhibitors to protect cells from HIV infection after 48 h of removal of drug from cell culture was 4'-Ed4T > LFD4C > DDI > D4T > 3TC > AZT > FTC > NVP. The composite ranking was obtained using previously published data on 4'-Ed4T, D4T, AZT, and NVP [11].

### Viral rebound after removal of drug from cell culture

Complex dosing schedules of antiretroviral therapy (ART) may lead to poor compliance and many patients take drug holidays for a few days to several weeks [21,22]. With the persistence of anti-HIV activity of 4'-Ed4T, LFD4C and DDI, we investigated if this will translate into delay in viral rebound after removal of inhibitor from HIV-infected cell culture (Figure 1B). This assay simulates a situation where a patient misses a couple of days of antiretroviral drug. TZM-bl cells pre-treated with different concentrations of an inhibitor were infected with HIV-1 IIIB virus at an MOI of 0.05 and incubated at 37°C. The culture media were changed to remove extracellular drug at 24, and 48 h post-infection without replacement of inhibitor and cells of a batch of the plates harvested at each time point. Luciferase activity was determined as a function of viral replication; luciferase activity similar to that of day one of the untreated virus control was considered as viral rebound. When the culture was treated with 5 × EC_50 _of the inhibitor, viral rebound occurred within 24 h with D4T, 3TC, NVP, and AZT (Table 2). Viral rebound was observed in cultures treated with 5 × EC_50 _of FTC and LFD4C at 48 h. With 4'-Ed4T and DDI, viral rebound occurred after 48 and 72 h, respectively. We observed a dose-dependent delay of viral rebound after removal of inhibitor from culture with 4'-Ed4T, LFD4C, D4T, NVP and 3TC. Interestingly, AZT did not delay viral rebound beyond 24 h even at 100 × EC_50 _(data not shown). In general, inhibitors that ranked high in the ability to protect cells from HIV infection were superior in preventing viral rebound after removal from cell culture. None of the inhibitors could completely prevent viral rebound after removal from culture. This suggests that there is always residual viral replication or the inhibition is reversible [19].

**Table 2:** Time to viral rebound to the level of untreated virus control at 24 h

**Inhibitor**	**Viral rebound (hours post wash)**^**a**^	
	5 × EC_50_^b^	50 × EC_50_^b^
4'-Ed4T	>48	>>72
LFD4C	48	n.d
DDI	>72	n.d
D4T	<24	>72
FTC	48	n.d
3TC	<24	48
NVP	<24	<24
AZT	<24	<24

^a ^Luciferase activity similar to that on day 1 of the untreated virus control was considered as viral rebound.

^b^EC_50 _based on values without preincubation.

n.d (not determined): The inhibitors were not tested at these concentrations either because of known cytotoxicity or unachievable concentration during therapy of HIV-infected individuals.

Results are the average of at least three independent experiments.

The antiviral activity of an inhibitor follows concentration-dependent kinetics. The serum concentration should stay well above the EC_50 _during the dosing interval for efficacy. We, therefore, assessed the fold-increase in EC_50 _of an inhibitor required to keep viral rebound at 50% of the untreated control after removal of drug for 48 and 72 h (Table 3). At 48 h after removal of drug, the fold-change in concentration of inhibitor required to keep viral rebound at 50% was in the order of DDI < 4'-Ed4T < LFD4C < FTC < D4T < 3TC < NVP < AZT. The fold-increase in EC_50 _at 72 h showed a similar order (Table 3). NVP, 3TC, and AZT required greater than 75, 150 and 1000 fold-increase, respectively, to maintain viral rebound at 50% of the untreated control. DDI, 4'- Ed4T, LFD4C and FTC were superior in preventing viral rebound.

**Table 3:** Fold-increase in EC_50 _required to keep viral growth at 50% over time

**Inhibitor**	**Fold-increase in EC_**50**_^**a **^(± S.D)**	
	48 h	72 h
4'-Ed4T	2.6 ± 0.4	8.6 ± 1.4
LFD4C	4.1 ± 1.2	13.5 ± 3.7
DDI	1.8 ± 0.5	2.5 ± 0.1
D4T	11.0 ± 1.2	38.4 ± 4.2
FTC	5.6 ± 0.9	>12.5^b^
3TC	17.3 ± 4.2	>75^b^
NVP	70.5 ± 14.1	>150^b^
AZT	127.8 ± 19.2	>1000^b^

^a^EC_50 _based on values without pre-incubation of the inhibitor as reported in Table 1, column 2.

^b ^Maximum concentration tested at 72 h.

### Persistence ranking of reverse transcriptase inhibitors

The protection of cells from HIV infection and the viral rebound assays were complementary and are measures of the persistence of an inhibitor after removal from cell culture. The persistence of anti-HIV activity is a function of an inhibitor's ability to protect cells from HIV infection and to delay viral rebound after removal from culture. Based on the two-component assay we ranked the inhibitors in the order of decreasing persistence of anti-HIV activity: DDI > 4'-Ed4T > LFD4C > FTC = D4T > 3TC > NVP > AZT.

## Discussion

The persistence of anti-HIV activity of an inhibitor presents a new way of looking at the pharmacodynamic property of an inhibitor in vitro that may predict its in vivo performance. It could complement other in vitro measures like EC_50_, inhibition quotient (IQ), and the recently described instantaneous inhibitory potential (IIP) [10], especially with regard to nucleoside analogs. Our two-component persistence assay combines measures of single viral replication-cycle and cumulative inhibition at multiple time-points. It is a function of an inhibitor's ability to protect cells from HIV infection and delay viral rebound after removal of inhibitor from cell culture. Inhibitors such as DDI, 4'-Ed4T, LFD4C, FTC, and D4T were superior in delaying viral rebound after removal from cell culture. The persistence of anti-HIV activity of an inhibitor is dependent on the intracellular pool of the inhibitor and its metabolites. Therefore, the rank order of the inhibitors should remain the same regardless of the cell type used. There may be inter-class differences but no significant intra-class differences, e.g., the order within the thymidine analogs should be reproducible across different cell lines. The NRTIs undergo stepwise phosphorylation by intracellular kinases to their active triphosphate metabolites; the triphosphate metabolites inhibit viral DNA synthesis by competing with natural dNTPs as substrate for viral DNA polymerase [2]. The efficiency of this process will depend on the expression and quality of the cellular kinases, intracellular dNTP pool, and the analog. For instance, different thymidine analogs have varying affinities to thymidine kinase (TK1) [23]. Moreover, the intracellular dNTP level varies significantly between different cell types and fluctuates during the cell cycle [24]. The pool of naturally occurring dNTPs is about 20-fold higher in established T-cell lines compared to that of primary cells [25]. There are individual differences in cellular transport and metabolism of NTRIs [26]. Taken together, our findings using transformed cell line may not be extrapolated to primary cells (PBMCs). However, our conditions are sufficient to allow us to observe differences in the persistence of anti-HIV activity of the inhibitors studied; our findings are, therefore, a reflection of the differences in the inhibitors. Though, the use of PBMCs for these studies will not be trivial, further studies using PBMCs are needed to validate the assay and the concept of persistent index.

Traditionally, the plasma concentration of inhibitors has been used as surrogate for designing dosage and monitoring HIV therapy [3,4]. However, plasma concentration of an inhibitor may not predict the intracellular concentration and the anti-HIV activity of an inhibitor [3,4,11,27]. The plasma elimination t_1/2 _of AZT, D4T, 4'-Ed4T, 3TC, FTC, tenofovir (TDF), and abacavir (ABC) are 0.5–3, 0.8–1.5, 2.3 to 3.7, 5–7, 8–10, 12–17, and 0.9–2.2 h, respectively [28,29]. While the intracellular t_1/2 _of AZT, D4T, 3TC, FTC, TDF, and ABC in human PBMCs are 3–4, 7, 15–16, 29–56, 60->175, and 12–21 h, respectively. The intracellular concentration of an inhibitor is more predictive of the anti-HIV activity of an inhibitor [30]. In our study, inhibitors with longer intracellular half lives tended to have superior persistence of anti-HIV activity. Witvrouw et al. demonstrated that TDF was the only analog tested that could delay viral rebound for 2 to 3 days after removal from cell culture [19]. The authors attributed the ability of TDF to delay viral rebound to its persistence in cells. This is consistent with an in vivo study where the TDF triphosphate concentration was still quantifiable in PBMCs of 6 of 8 and 2 of 8 patients 14 and 28 days after the last dose of TDF, respectively [31]. The triphosphate concentration of ABC in patients fell to below the limit of detection in all patients studied by 72 h after the last ABC dose. Thus, the persistence assay is a function of the intracellular concentration. Moore et al. found a linear relationship between intracellular concentration of AZT-TP and 3TC-TP and changes in plasma HIV-1 RNA concentration [30]. Our findings are consistent with the findings of the in vitro and in vivo studies reported above. Our persistence assay is a surrogate measure of intracellular concentration and anti-HIV activity of an inhibitor. Moreover, the inhibitors were studied using the same cell line and experimental conditions affording us the ability to compare the persistence of anti-HIV activity of one inhibitor to the other. Furthermore, the assay could be explored as a cost-effective measure of intracellular concentration.

The persistence of anti-HIV activity could have several clinical applications; selection of nucleoside analog backbone for combination antiretroviral therapy, selection of appropriate pre- and post exposure prophylaxis (PrEP and PEP), and prediction of drugs that may be more "forgiving" if a patient inadvertently takes drug holidays. Most current HAART regimens comprise of two nucleoside analogs and a non nucleoside analog or a protease inhibitor [1]. Therefore, NRTIs continue to be an important part of HAART. Shen et al. found that the IIP of NRTIs were ≤ 3.5 and generally less than that of NNRTI and PIs [10]. The use of IIP to select an NRTI backbone may not be discriminatory enough. Inhibitors that do not require intracellular activation for antiviral activity tended to have low IIP values. Therefore, the persistence of anti-HIV activity assay may be more sensitive and discriminatory for the selection of NRTI backbone of HAART. FTC delayed viral rebound better than 3TC; fixed-dose combination with tenofovir (TDF) has demonstrated superior efficacy in clinical practice. The ability of FTC to delay viral rebound is comparable to that of DDI, 4'-Ed4T, LFD4C and TDF. Combination of two NRTIs with better persistence of anti-HIV activity may be an ideal NRTI backbone for HAART. 4'-Ed4T, currently in clinical development, in combination with cytidine analogs has shown synergistic anti-HIV activity in vitro [13]. The persistence of antiviral activity of 4'-Ed4T after removal of drug from culture may be due to; 1) the fact that 4'-Ed4TTP once formed remains relatively stable and active in cells, and that the pool of 4'-Ed4TMP may continue to replenish the critical concentration of 4'-Ed4TTP, 2) less efficient removal of incorporated nucleotide by exonucleases from terminal viral DNA and 3) inability of 4'-Ed4T metabolites to permeate the cell membrane by non-facilitated diffusion (unpublished data) as compared to the metabolites of AZT [32]. If proven to have favorable safety profile, and efficacious in clinical trials, it could be co-formulated with a cytidine analog as HAART backbone.

Also, combination of the persistence of anti-HIV activity and potency will have useful clinical applications. For instance, DDI, at the current clinical dosing, is associated with pancreatitis as a side effect [33]. DDI in our assay was the best in delaying viral rebound. It is possible that its persistence in certain cells (e.g., pancreatic cells) may be responsible for the observed clinical side effects. It has been observed that rates of pancreatitis in HIV-infected individuals on DDI seem to have positive correlation with dosage [33]. Therefore, the combination of the persistence of anti-HIV activity and potency may be useful in selecting a dosage that is adequate to achieve viral suppression while avoiding side effects (e.g., pancreatitis).

The drug persistence assay simulates the concept of HIV pre- and post- exposure prophylaxis. Animal models show that after initial exposure, HIV replicates within dendritic cells of the skin and mucosa before spreading through lymphatic vessels and developing into a systemic infection [34]. The delay in the viremic phase offers a "window of opportunity" for PEP using antiretroviral drugs [35,36]. An antiretroviral agent with persistence of anti-HIV activity even after its removal will be ideal for PrEP or PEP. For instance, TDF with prolong intracellular half life and ability to delay viral rebound has been found to be a useful antiviral agent for PEP and it is being evaluated in clinical trials for PrEP [37-39]. Therefore, 4'-Ed4T and LFD4C, which are still in clinical development, are promising candidate antiviral agents for PrEP and PEP.

Residual HIV viral replication is often observed even with currently effective drugs in treatment-experienced individuals with no signs of overt disease [40,41]. An inhibitor with a superior persistence of anti-HIV activity may be more "forgiving" when a patient inadvertently misses a couple of doses. We simulated a scenario where patients may give themselves drug holidays for a few days (e.g., due to intercurrent illness, surgery, toxicity or unavailable medication). The culture media were changed to remove extracellular drug at 24, and 48 h post-infection without replacement of inhibitor to simulate 24, and 48 h of drug holiday, respectively. Inhibitors with longer intracellular half-lives and/or less efficiently removed from the terminal of HIV DNA delayed viral rebound longer [17,42]. Consistent with clinical observations, the time to viral rebound correlated positively with the efficacy of the inhibitor [42-44]. None of the inhibitors studied was able to completely prevent viral rebound. This is consistent with previous findings of the removal of drug pressure in short-term in vitro assays [19]. In contrast, there have been reports of complete clearance of HIV from virus-infected cells after prolonged subcultivations [45,46]. Viral rebound occurred within 24 h with most of the antiretroviral agents currently in clinical use (AZT, D4T, 3TC, and NVP) at 5 × EC_50 _concentrations (Table 2). With FTC and LFD4C, cytidine analogs with longer intracellular half-lives than 3TC, viral rebound occurred at 48 h after removal of drug from culture. All the inhibitors, except AZT and NVP, exhibited a dose-dependent delay in viral rebound. Our findings of the inability of AZT to prevent viral rebound is consistent with previous studies where AZT at 500 ×-, and 1000 × EC_50 _failed to prevent viral breakthrough beyond 24 h [19,45]. Contrary to our expectation, NVP, which is used as a single dose to prevent mother-to-child transmission of HIV because of its favorable pharmacokinetics (t_1/2 _of 30 h) [47], did not delay viral rebound at 50 × EC_50_. High prevalence of NVP resistance mutations (e.g., K103N and Y181C) has been reported among mothers and infants who received a single dose NVP to prevent vertical transmission of HIV-1 [48]. Though, NVP had long plasma half-life, our findings may suggest that the rapid evolution of NVP resistant mutation is probably due to its poor intracellular retention. Based on the EC_50_, 3TC was about 2-fold potent than FTC, however, the anti-HIV activity of FTC persisted longer than 3TC. Therefore, the potency of an analog based on the EC_50 _may not be adequate to predict its activity. FTC-TP is incorporated about10-fold more efficiently than 3TC-TP by HIV-1 RT during RNA-dependent DNA synthesis [49]. The degree of viral rebound may depend on residual viral activity and the rate of removal of the incorporated triphosphate from the viral DNA chain to allow incorporation of natural dNTP. The delay in viral rebound with FTC treatment may be due to the slow excision of incorporated triphosphate of FTC in comparison with that of 3TC [49].

While we must exercise caution in drawing conclusions on clinical effects based on data from in vitro experiments, recent studies have demonstrated that current assays may not be adequate in predicting in vitro performance of an inhibitor and therefore a need for better assays. Ferguson et al. in a mathematical model, suggested that the best in vitro assay to reflect in vivo effect should be based on either single replication-cycle assays or a measure of cumulative inhibition at multiple time points [9]. Our two-component persistence assay satisfies the above criteria and was able to detect differences in the persistence of anti-HIV activity of inhibitors tested.

## Conclusion

The persistence of anti-HIV activity of the inhibitors (table 4) was derived from assays based on measures of single viral replication-cycle and cumulative inhibition at multiple time-points and therefore a better indicator of the pharmacodynamic property of an inhibitor. The persistence assay may complement EC_50_, IQ, and IIP in the selection of combination regimen (e.g., the NTRI-backbone) and better predict clinical effect of novel inhibitors. In addition, the discovery of novel drugs with superior persistence of anti-HIV activity may change viral dynamics in treatment-experienced individuals. Further studies using primary cells to validate our findings are needed.

**Table 4:** Persistence index and ranking of HIV inhibitors

**Inhibitor**	^**a**^**EC**_**50**_**(μM)**	^**b**^**EC**_**50**_**(μM)**	**Persistence Index (Pi)** (^a^EC_50_/^b^EC_50_)
DDI	18.6 ± 5.4	5.4 ± 0.8	3.4
4'-Ed4T	2.7 ± 0.1	0.37 ± 0.15	7.3
LFD4C	1.4 ± 0.4	0.16 ± 0.08	8.8
D4T	11.9 ± 1.0	1.30 ± 0.21	9.2
FTC	12.0 ± 3.0	1.03 ± 0.23	11.6
3TC	6.93 ± 2.87	0.53 ± 0.01	13.1
NVP	1.41 ± 0.85	0.063 ± 0.04	22.4
AZT	2.3 ± 1.6	0.074 ± 0.04	31.1

^a ^Concentration required to keep viral rebound at 50% for 24 h after removal of inhibitor

^b ^Concentration required to protect 50% of cells from infection for 24 h after removal of inhibitor

## Competing interests

Y-CC is a co-inventor of L-FD4C and 4'-Ed4T as anti-HIV compounds.

## Authors' contributions

EP: Designed the study, participated in the experiments, interpreted and analyzed the data, and drafted the manuscript. SPG: Designed the study, participated in the experiments, interpreted and analyzed the data, and reviewed the drafted manuscript. GED: Designed the study, participated in the experiments, interpreted and analyzed the data, and reviewed the drafted manuscript. YCC: Designed the study, supervised the experiments, interpreted the data, and reviewed the drafted manuscript. All the authors read and approved the final manuscript.

## References

[B1] (2008). Panel on Antiretroviral Guidelines for Adult and Adolescents. Guidelines for the use of antiretroviral agents in HIV-1-infected adults and adolescents. Department of Health and Human Services. http://aidsinfo.nih.gov/Guidelines.

[B2] Kakuda TN (2000). Pharmacology of nucleoside and nucleotide reverse transcriptase inhibitor-induced mitochondrial toxicity. Clin Ther.

[B3] Back D, Gatti G, Fletcher C, Garaffo R, Haubrich R, Hoetelmans R, Kurowski M, Luber A, Merry C, Perno CF (2002). Therapeutic drug monitoring in HIV infection: current status and future directions. AIDS.

[B4] Gerber JG, Acosta EP (2003). Therapeutic drug monitoring in the treatment of HIV-infection. J Clin Virol.

[B5] Spenlehauer C, Gordon CA, Trkola A, Moore JP (2001). A luciferase-reporter gene-expressing T-cell line facilitates neutralization and drug-sensitivity assays that use either R5 or X4 strains of human immunodeficiency virus type 1. Virology.

[B6] Hachiya A, Aizawa-Matsuoka S, Tanaka M, Takahashi Y, Ida S, Gatanaga H, Hirabayashi Y, Kojima A, Tasumi M, Oka S (2001). Rapid and simple phenotypic assay for drug susceptibility of human immunodeficiency virus type 1 using CCR5-expressing HeLa/CD4(+) cell clone 1–10 (MAGIC-5). Antimicrob Agents Chemother.

[B7] Gervaix A, West D, Leoni LM, Richman DD, Wong-Staal F, Corbeil J (1997). A new reporter cell line to monitor HIV infection and drug susceptibility in vitro. Proc Natl Acad Sci USA.

[B8] Chiba-Mizutani T, Miura H, Matsuda M, Matsuda Z, Yokomaku Y, Miyauchi K, Nishizawa M, Yamamoto N, Sugiura W (2007). Use of new T-cell-based cell lines expressing two luciferase reporters for accurately evaluating susceptibility to anti-human immunodeficiency virus type 1 drugs. J Clin Microbiol.

[B9] Ferguson NM, Fraser C, Anderson RM (2001). Viral dynamics and anti-viral pharmacodynamics: rethinking in vitro measures of drug potency. Trends Pharmacol Sci.

[B10] Shen L, Peterson S, Sedaghat AR, McMahon MA, Callender M, Zhang H, Zhou Y, Pitt E, Anderson KS, Acosta EP, Siliciano RF (2008). Dose-response curve slope sets class-specific limits on inhibitory potential of anti-HIV drugs. Nat Med.

[B11] Paintsil E, Dutschman GE, Hu R, Grill SP, Lam W, Baba M, Tanaka H, Cheng YC (2007). Intracellular metabolism and persistence of the anti-human immunodeficiency virus activity of 2',3'-didehydro-3'-deoxy-4'-ethynylthymidine, a novel thymidine analog. Antimicrob Agents Chemother.

[B12] Wei X, Decker JM, Liu H, Zhang Z, Arani RB, Kilby JM, Saag MS, Wu X, Shaw GM, Kappes JC (2002). Emergence of resistant human immunodeficiency virus type 1 in patients receiving fusion inhibitor (T-20) monotherapy. Antimicrob Agents Chemother.

[B13] Dutschman GE, Grill SP, Gullen EA, Haraguchi K, Takeda S, Tanaka H, Baba M, Cheng YC (2004). Novel 4'-substituted stavudine analog with improved anti-human immunodeficiency virus activity and decreased cytotoxicity. Antimicrob Agents Chemother.

[B14] Haraguchi K, Takeda S, Tanaka H, Nitanda T, Baba M, Dutschman GE, Cheng YC (2003). Synthesis of a highly active new anti-HIV agent 2',3'-didehydro-3'-deoxy-4'-ethynylthymidine. Bioorg Med Chem Lett.

[B15] Waheed AA, Ablan SD, Mankowski MK, Cummins JE, Ptak RG, Schaffner CP, Freed EO (2006). Inhibition of HIV-1 replication by amphotericin B methyl ester: selection for resistant variants. J Biol Chem.

[B16] Lin TS, Luo MZ, Liu MC, Zhu YL, Gullen E, Dutschman GE, Cheng YC (1996). Design and synthesis of 2',3'-dideoxy-2',3'-didehydro-beta-L-cytidine (beta-L-d4C) and 2',3'-dideoxy 2',3'-didehydro-beta-L-5-fluorocytidine (beta-L-Fd4C), two exceptionally potent inhibitors of human hepatitis B virus (HBV) and potent inhibitors of human immunodeficiency virus (HIV) in vitro. J Med Chem.

[B17] Dutschman GE, Bridges EG, Liu SH, Gullen E, Guo X, Kukhanova M, Cheng YC (1998). Metabolism of 2',3'-dideoxy-2',3'-didehydro-beta-L(-)-5-fluorocytidine and its activity in combination with clinically approved anti-human immunodeficiency virus beta-D(+) nucleoside analogs in vitro. Antimicrob Agents Chemother.

[B18] Bridges EG, Dutschman GE, Gullen EA, Cheng YC (1996). Favorable interaction of beta-L(-) nucleoside analogues with clinically approved anti-HIV nucleoside analogues for the treatment of human immunodeficiency virus. Biochem Pharmacol.

[B19] Witvrouw M, Pannecouque C, Desmyter J, De Clercq E, Andries K (2000). In vitro evaluation of the effect of temporary removal of HIV drug pressure. Antiviral Res.

[B20] Moore KH, Barrett JE, Shaw S, Pakes GE, Churchus R, Kapoor A, Lloyd J, Barry MG, Back D (1999). The pharmacokinetics of lamivudine phosphorylation in peripheral blood mononuclear cells from patients infected with HIV-1. AIDS.

[B21] Podsadecki TJ, Vrijens BC, Tousset EP, Rode RA, Hanna GJ (2007). Decreased adherence to antiretroviral therapy observed prior to transient human immunodeficiency virus type 1 viremia. J Infect Dis.

[B22] Gardner EM, Sharma S, Peng G, Hullsiek KH, Burman WJ, Macarthur RD, Chesney M, Telzak EE, Friedland G, Mannheimer SB (2008). Differential adherence to combination antiretroviral therapy is associated with virological failure with resistance. AIDS.

[B23] Balzarini J (1994). Metabolism and mechanism of antiretroviral action of purine and pyrimidine derivatives. Pharm World Sci.

[B24] Meyerhans A, Vartanian JP, Hultgren C, Plikat U, Karlsson A, Wang L, Erikson S, Wain-Hobson S (1994). Restriction and enhancement of human immunodeficiency virus type 1 replication by modulation of intracellular deoxynucleoside triphosphate pools. J Virol.

[B25] Back NK, Nijhuis M, Keulen W, Boucher CA, Oude Essink BO, van Kuilenburg AB, van Gennip AH, Berkhout B (1996). Reduced replication of 3TC-resistant HIV-1 variants in primary cells due to a processivity defect of the reverse transcriptase enzyme. Embo J.

[B26] Anderson PL, Kakuda TN, Kawle S, Fletcher CV (2003). Antiviral dynamics and sex differences of zidovudine and lamivudine triphosphate concentrations in HIV-infected individuals. AIDS.

[B27] Moyer TP, Temesgen Z, Enger R, Estes L, Charlson J, Oliver L, Wright A (1999). Drug Monitoring of Antiretroviral Therapy for HIV-1 Infection: Method Validation and Results of a Pilot Study. Clin Chem.

[B28] Schinazi RF, Hernandez-Santiago BI, Hurwitz SJ (2006). Pharmacology of current and promising nucleosides for the treatment of human immunodeficiency viruses. Antiviral Res.

[B29] Mastuda T, Paintsil E, Ross JS, Schofield J, Cheng YC, Urata Y (2009). A Single Dose Escalation Study to Evaluate the Safety, Tolerability, and Pharmacokinetics of OBP-601 (4;-Ed4Ta Novel NRTI) in Healthy Subjects. 16th Conference on Retroviruses and Opportunistic Infections Montreal, Canada.

[B30] Moore JD, Acosta EP, Johnson VA, Bassett R, Eron JJ, Fischl MA, Long MC, Kuritzkes DR, Sommadossi JP (2007). Intracellular nucleoside triphosphate concentrations in HIV-infected patients on dual nucleoside reverse transcriptase inhibitor therapy. Antivir Ther.

[B31] Hawkins T, Veikley W, St Claire RL, Guyer B, Clark N, Kearney BP (2005). Intracellular pharmacokinetics of tenofovir diphosphate, carbovir triphosphate, and lamivudine triphosphate in patients receiving triple-nucleoside regimens. J Acquir Immune Defic Syndr.

[B32] Zimmerman TP, Mahony WB, Prus KL (1987). 3'-azido-3'-deoxythymidine. An unusual nucleoside analogue that permeates the membrane of human erythrocytes and lymphocytes by nonfacilitated diffusion. J Biol Chem.

[B33] Reisler RB, Murphy RL, Redfield RR, Parker RA (2005). Incidence of pancreatitis in HIV-1-infected individuals enrolled in 20 adult AIDS clinical trials group studies: lessons learned. J Acquir Immune Defic Syndr.

[B34] Tsai CC, Emau P, Follis KE, Beck TW, Benveniste RE, Bischofberger N, Lifson JD, Morton WR (1998). Effectiveness of postinoculation (R)-9-(2-phosphonylmethoxypropyl) adenine treatment for prevention of persistent simian immunodeficiency virus SIVmne infection depends critically on timing of initiation and duration of treatment. J Virol.

[B35] Bottiger D, Johansson NG, Samuelsson B, Zhang H, Putkonen P, Vrang L, Oberg B (1997). Prevention of simian immunodeficiency virus, SIVsm, or HIV-2 infection in cynomolgus monkeys by pre- and postexposure administration of BEA-005. AIDS.

[B36] Smith DK, Grohskopf LA, Black RJ, Auerbach JD, Veronese F, Struble KA, Cheever L, Johnson M, Paxton LA, Onorato IM, Greenberg AE (2005). Antiretroviral postexposure prophylaxis after sexual, injection-drug use, or other nonoccupational exposure to HIV in the United States: recommendations from the U.S. Department of Health and Human Services. MMWR Recomm Rep.

[B37] Cranage M, Sharpe S, Herrera C, Cope A, Dennis M, Berry N, Ham C, Heeney J, Rezk N, Kashuba A, Anton P, McGowan I, Shattock R (2008). Prevention of SIV rectal transmission and priming of T cell responses in macaques after local pre-exposure application of tenofovir gel. PLoS Med.

[B38] Dumond JB, Yeh RF, Patterson KB, Corbett AH, Jung BH, Rezk NL, Bridges AS, Stewart PW, Cohen MS, Kashuba AD (2007). Antiretroviral drug exposure in the female genital tract: implications for oral pre- and post-exposure prophylaxis. AIDS.

[B39] Chi BH, Sinkala M, Mbewe F, Cantrell RA, Kruse G, Chintu N, Aldrovandi GM, Stringer EM, Kankasa C, Safrit JT, Stringer JS (2007). Single-dose tenofovir and emtricitabine for reduction of viral resistance to non-nucleoside reverse transcriptase inhibitor drugs in women given intrapartum nevirapine for perinatal HIV prevention: an open-label randomised trial. Lancet.

[B40] Sharkey ME, Teo I, Greenough T, Sharova N, Luzuriaga K, Sullivan JL, Bucy RP, Kostrikis LG, Haase A, Veryard C, Davaro RE, Cheesman SH, Daly JS, Bova C, Ellison RT, Mady B, Lai KK, Moyle G, Nelson M, Gazzard B, Shaunak S, Stevenson M (2000). Persistence of episomal HIV-1 infection intermediates in patients on highly active anti-retroviral therapy. Nat Med.

[B41] Ramratnam B, Mittler JE, Zhang L, Boden D, Hurley A, Fang F, Macken CA, Perelson AS, Markowitz M, Ho DD (2000). The decay of the latent reservoir of replication-competent HIV-1 is inversely correlated with the extent of residual viral replication during prolonged anti-retroviral therapy. Nat Med.

[B42] Nakata H, Amano M, Koh Y, Kodama E, Yang G, Bailey CM, Kohgo S, Hayakawa H, Matsuoka M, Anderson KS, Cheng YC, Mitsuya H (2007). Activity against human immunodeficiency virus type 1, intracellular metabolism, and effects on human DNA polymerases of 4'-ethynyl-2-fluoro-2'-deoxyadenosine. Antimicrob Agents Chemother.

[B43] Bedimo R, Chen RY, Westfall AO, Raper JL, Allison JJ, Saag MS (2006). Sustained HIV viral suppression following treatment interruption: an observational study. AIDS Res Hum Retroviruses.

[B44] Eron JJ, Bartlett JA, Santana JL, Bellos NC, Johnson J, Keller A, Kuritzkes DR, St Clair MH, Johnson VA (2004). Persistent antiretroviral activity of nucleoside analogues after prolonged zidovudine and lamivudine therapy as demonstrated by rapid loss of activity after discontinuation. J Acquir Immune Defic Syndr.

[B45] Balzarini J, Karlsson A, Perez-Perez MJ, Camarasa MJ, De Clercq E (1993). Knocking-out concentrations of HIV-1-specific inhibitors completely suppress HIV-1 infection and prevent the emergence of drug-resistant virus. Virology.

[B46] Okamoto M, Makino M, Yamada K, Nakade K, Yuasa S, Baba M (1996). Complete inhibition of viral breakthrough by combination of MKC-442 with AZT during a long-term culture of HIV-1 infected cells. Antiviral Res.

[B47] Cheeseman SH, Hattox SE, McLaughlin MM (1993). Pharmacokinetics of nevirapine: initial single-rising-dose study in humans. Antimicrob Agents Chemother.

[B48] Eshleman SH, Mracna M, Guay LA, Deseyve M, Cunningham S, Mirochnick M, Musoke P, Fleming T, Glenn Fowler M, Mofenson LM, Mmiro F, Jackson JB (2001). Selection and fading of resistance mutations in women and infants receiving nevirapine to prevent HIV-1 vertical transmission (HIVNET 012). AIDS.

[B49] Feng JY, Murakami E, Zorca SM, Johnson AA, Johnson KA, Schinazi RF, Furman PA, Anderson KS (2004). Relationship between antiviral activity and host toxicity: comparison of the incorporation efficiencies of 2',3'-dideoxy-5-fluoro-3'-thiacytidine-triphosphate analogs by human immunodeficiency virus type 1 reverse transcriptase and human mitochondrial DNA polymerase. Antimicrob Agents Chemother.

